# Single-cell sequencing and transcriptome analyses in the construction of a liquid–liquid phase separation-associated gene model for rheumatoid arthritis

**DOI:** 10.3389/fgene.2023.1210722

**Published:** 2023-10-25

**Authors:** Jiaojiao Tai, Linbang Wang, Ziqiang Yan, Jingkun Liu

**Affiliations:** ^1^ Department of Orthopedics, Honghui Hospital, Xi’an Jiaotong University, Xi’an, Shaanxi, China; ^2^ Department of Orthopedics, Peking University Third Hospital, Beijing, China

**Keywords:** rheumatoid arthritis, liquid-liquid phase separation, bioinformatics, transcriptome, single-cell sequencing, MYC, MAP1LC3B

## Abstract

**Background:** Rheumatoid arthritis (RA) is a disabling autoimmune disease that affects multiple joints. Accumulating evidence suggests that imbalances in liquid–liquid phase separation (LLPS) can lead to altered spatiotemporal coordination of biomolecular condensates, which play important roles in carcinogenesis and inflammatory diseases. However, the role of LLPS in the development and progression of RA remains unclear.

**Methods:** We screened RA and normal samples from GSE12021, GSE55235, and GSE55457 transcriptome datasets and GSE129087 and GSE109449 single-cell sequencing datasets from Gene Expression Omnibus database to investigate the pathogenesis of LLPS-related hub genes at the transcriptome and single cell sequencing levels. Machine learning algorithms and weighted gene co-expression network analysis were applied to screen hub genes, and hub genes were validated using correlation studies.

**Results:** Differential analysis showed that 36 LLPS-related genes were significantly differentially expressed in RA, further random forest and support vector machine identified four and six LLPS-related genes, respectively, and weighted gene co-expression network analysis identified 396 modular genes. Hybridization of the three sets revealed two hub genes, MYC and MAP1LC3B, with AUCs of 0.907 and 0.911, respectively. Further ROC analysis of the hub genes in the GSE55457 dataset showed that the AUCs of MYC and MAP1LC3B were 0.815 and 0.785, respectively. qRT-PCR showed that the expression of MYC and MAP1LC3B in RA synovial tissues was significantly lower than that in the normal control synovial tissues. Correlation analysis between hub genes and the immune microenvironment and single-cell sequencing analysis revealed that both MYC and MAP1LC3B were significantly correlated with the degree of infiltration of various innate and acquired immune cells.

**Conclusion:** Our study reveals a possible mechanism for LLPS in RA pathogenesis and suggests that MYC and MAP1LC3B may be potential novel molecular markers for RA with immunological significance.

## 1 Introduction

Rheumatoid arthritis (RA) is an autoimmune disease involving multiple joints that is characterized by tenosynovitis, resulting in both cartilage destruction and bone erosion. Until the 1990s, RA frequently resulted in disability, inability to work, and increased mortality. Newer treatment options made RA a manageable disease (Lin et al., 2020). In recent years, an increasing number of recent studies have demonstrated that the innate immune system plays a critical role in the development and progression of RA and that a variety of innate immune cells, including monocytes, macrophages, dendritic cells, autoreactive CD4^+^ T cells and pathogenic B cells, are involved in the inflammatory response in RA patients, which in turn activates the adaptive immune system (Edilova et al., 2021; Jang et al., 2022).

Liquid–liquid phase separation (LLPS) (Falahati and Haji-Akbari, 2019) is a recent discovery in molecular cell biology. It is a reversible condensate generation-driven process that generates membraneless organelles (MLOs) that exert pan-cellular functions under normal conditions and stress (Alberti, 2017; Cobos et al., 2018; Alberti et al., 2019; Pancsa et al., 2019). The functional advantage of MLOs does not arise directly from the individual actions of their constituent molecules but rather from their collective behavior (Hamill et al., 2002; Lee et al., 2013; Alberti et al., 2019; Pancsa et al., 2019). Although early studies of abnormal LLPS processes and lectin formation focused on specific neurodegenerative diseases, emerging research targeting LLPS has received increasing attention in the field of cancer, where it has been found that LLPS can alter the normal function of oncogenes or antioncogenes, thereby promoting tumorigenesis and progression (Zbinden et al., 2020; Quiroga et al., 2022). Also, LLPS can promote tumor progression by regulating tumor-related signaling pathways (Peng et al., 2022). Numerous studies have shown that LLPS not only be involved in the pathological process of type 2 diabetes and metabolic bone disease, but also promote virus-induced inflammation (Wu et al., 2021; Chen et al., 2022). Phase separation has been shown to play a role in immune signaling such as T cell receptor, B cell receptor, cyclic GMP–AMP synthase, retinoic acid-inducible gene I protein and nuclear factor-κB pathways (Xiao et al., 2022). The study of LLPS-mediated regulation of biological processes remains in its early stages, and a better understanding of the molecular mechanisms involved and their impact on cells and organisms is needed.

Studies into the immunopathogenesis of RA have spanned decades. RA is now understood as a highly heterogeneous chronic immune-mediated disease, in which multiple immune cell types and signaling networks are dysfunctional, resulting in maladaptation (Bartok and Firestein, 2010; Jiang et al., 2021). Although the role of LLPS in RA has not been reported, it has been suggested that LLPS-derived protein aggregates are responsible for age-related diseases, and that if molecular condensates formed through LLPS cannot be tightly controlled, they can lead to protein misfolding and aggregation, further contributing to the progression of aging-related diseases (Alberti and Hyman, 2021). Meanwhile, LLPS has been found to mediate both innate and adaptive immune responses (Du and Chen, 2018; Wang et al., 2021), while RA is considered a typical autoimmune disease and immunosenescence can exacerbate joint discomfort in patients with RA (Barbé-Tuana et al., 2020). Therefore, this study combines LLPS with RA for the first time to explore the role of LLPS in RA. This study aimed to investigate the role of LLPS-related genes and immune pathogenic mechanisms in RA lesions using transcriptome data and establish a model to predict the prognosis and immune status of patients with RA. We identified biomarkers of LLPS-related genes in the disease process and explored the immune mechanisms by which these markers may be involved. Overall, our results reveal insights into new therapeutic concepts and biomarkers for RA.

## 2 Materials and methods

### 2.1 Acquisition of data and screening of differentially expressed genes (DEGs)

Gene expression profile data for human synovial tissues were obtained from the GSE12021 (Huber et al., 2008) (https://www.ncbi.nlm.nih.gov/geo/query/acc.cgi?acc=GSE12021), GSE55235 (Woetzel et al., 2014) (https://www.ncbi.nlm.nih.gov/geo/query/acc.cgi?acc=GSE55235), and GSE55457 (Woetzel et al., 2014) (https://www.ncbi.nlm.nih.gov/geo/query/acc.cgi?acc=GSE55457) datasets of the Gene Expression Omnibus (GEO) database (Barrett et al., 2007). Twelve RA samples and nine normal samples from the GPL96 ([HG-U133A] Affymetrix Human Genome U133A Array) sequencing platform of the GSE12021 dataset, ten RA samples and ten normal samples from the GPL96 [(HG-U133A) Affymetrix Human Genome U133A Array] sequencing platform of the GSE55235 dataset, and thirteen RA samples and ten normal samples from the GPL96 [(HG-U133A) Affymetrix Human Genome U133A Array] sequencing platform of the GSE55457 dataset were used in this study. We used the combat function of the R software “sva” package (https://bioconductor.org/packages/release/bioc/html/sva.html) (Leek et al., 2012) to pre-process the data sets expression matrix of GSE12021 and GSE55235 datasets, including data background adjustment, normalization, and merging.

### 2.2 Panoramic analysis of LLPS-related genes in RA

LLPS-related genes were selected from PhaSepDB (Hou et al., 2022), an online database that records all LLPS-related genes (http://db.phasep.pro). We first used the Perl language to extract LLPS-related gene expression data and applied the limma (Ritchie et al., 2015) package in the R language to screen LLPS-related DEGs from RA and normal synovium using the screening condition *p* < 0.05. In addition, we performed a co-expression analysis of these DEGs and visualized gene relationship pairs with correlation coefficients greater than 0.4. We also constructed an LLPS-related DEGs interaction network using the network analysis R (Theodosiou et al., 2017) package. Finally, we observed the chromosomal localization of LLPS-related DEGs, which were visualized using the R circos (An et al., 2015) package.

### 2.3 Molecular subtype classification of LLPS-related genes and functional enrichment analysis

We applied the “ConsensusClusterPlus” (Wilkerson and Hayes, 2010) (http://www.bioconductor.org/packages/release/bioc/html/ConsensusClusterPlus.html) package in R language, we classified RA samples into different sub molecular subtypes based on differential genes between RA and normal tissue (log2FC > 2.5, *p* < 0.05). Parameters were set to 50 replicates (reps = 50) and a resampling rate of 80% (pItem = 0.8). Finally, we performed functional enrichment analysis of the DEGs for the sub molecular types.

### 2.4 Machine learning methods and signature gene screening

This study first used two machine learning algorithms, random forest (RF) and support vector machine (SVM), to identify the characteristic genes of RA, and then applied the Weighted Gene Co-Expression Network Analysis (WGCNA) method to screen out modular genes that were significantly associated with LLPS.

RF is an integrated machine learning algorithm for feature screening of classification trees based on the Gini coefficient minimization criterion, which is highly adaptable to data, widely used, and has an outstanding advantage in gene identification of genomic data, and the algorithm also takes into account the correlations and interactions between features (Chen and Ishwaran, 2012; Speiser et al., 2019).

SVMs are widely used in pattern recognition and machine learning. Support vector machine recursive feature elimination (SVM-RFE) is a sequential inverse selection algorithm based on the maximum margin principle of SVM, which means that a model is used to train a sample, each feature is labeled and scored, the lowest scoring feature is removed, the remaining features are used again for model training, and so on, and the desired features are finally selected (Lin et al., 2012). The packages “e1071,” “kernlab,” and “caret” were used to eliminate the recursive features of the obtained DEGs and data calculation, and the best gene signature was obtained (Wang and Liu, 2015; Mahmoudian et al., 2021).

WGCNA is a widely employed approach for translating expression data into co-expression modules and investigating the relationships between modules and phenotypic traits (Yang et al., 2022). We first calculated the LLPS score of each sample based on the expression of LLPS-related genes in the samples using principal component analysis (Liu et al., 2021a), and calculated the stromal score of each sample using the ESTIMATE method (Yoshihara et al., 2013), and then clustered the genes with similar patterns based on the transcriptome profiles, stromal scores, and LLPS scores of the RA samples using the “WGCNA” R package (Langfelder and Horvath, 2008). In general, modules with high absolute values of module correlation were considered to have greater biological significance. Key criteria for module gene screening were gene significance >0.5, module membership >0.7, and *p* < 0.001.

The hub genes were obtained by crossing the three sets of genes screened by RF, SVM-RFE, and WGCNA. We then performed a correlation analysis between hub genes and LLPS-related genes.

### 2.5 Building and validating a predictive nomogram

Using the “rms” R package (Wells et al., 2018), we constructed a nomogram to assess risk for hub genes, the discriminatory power of the nomogram was validated by several methods. The performance of the nomogram was assessed by calibration and discrimination, and a calibration plot (1,000 bootstrap resamples) was used to evaluate the discrimination of the model. Harrell’s concordance index ranged from 0.5 (indicates absence of discrimination) to 1 (perfect discrimination) (Low et al., 2019), which is approximately equivalent to the receiver operating characteristic (ROC) area under curve (AUC). Furthermore, decision curve analysis (DCA) was employed to evaluate the clinical values and utility of the nomogram by R function “stdca” (Wang et al., 2020). Subsequently, we validated the accuracy and sensitivity of the hub genes in the GSE55457 dataset, constructed ROC curves for the hub genes, and calculated the AUC.

### 2.6 Single-cell sequencing analysis

We obtained serum transfer inflammatory arthritis and RA tissue single-cell RNA sequencing (scRNA-seq) data from the GSE129087 (Croft et al., 2019) (https://www.ncbi.nlm.nih.gov/geo/query/acc.cgi?acc=GSE129087) and GSE109449 (Mizoguchi et al., 2018) (https://www.ncbi.nlm.nih.gov/geo/query/acc.cgi?acc=GSE109449) dataset, respectively. Single-cell RNA-seq can provide RNA expression profiles for each cell independently, and differences in gene and protein expression levels can be observed on a single-cell basis (Wang et al., 2023). First, we selected the scRNA sequencing data in the GSE129087 dataset and performed data dimensionality reduction using the t-distributed random neighbor embedding (t-SNE) method to identify distinct cellular subpopulations. Subsequently, the scRNA sequencing data in the GSE109449 dataset further revealed distinct fibroblast subpopulations, and cell population classification, cell type identification, and cell differentiation trajectory analysis were performed using the “Seurat”, “SingleR”, and “Monocle” R software packages, respectively (Trapnell et al., 2014; Butler et al., 2018; Aran et al., 2019).

### 2.7 Validation of quantitative real-time polymerase chain reaction (qRT-PCR) for hub genes

The selection criterion for patients with RA in this study was based on the American Rheumatism Association 1987 revised classification criteria for RA. Synovial membranes of healthy controls were obtained from patients who underwent patellar repair surgery for patellar trauma (approval number: 202210006). All samples were obtained after obtaining informed consent from the patients. The samples were stored in RNAlater (Ambion) at −70°C. RNA was extracted from the synovial membranes using a UNIQ-10 Columnar Total RNA Purification Kit (Sangon Biotech, China). RNA quality and concentration was assessed using a SMA4000 microspectrophotometer (Merinton Instrument, Inc. MI, United States). The extracted RNA was reverse transcribed using an RR047A cDNA Synthesis Kit (TaKaRa, China). The qRT-PCR of pivotal genes was performed using 2X SG Fast qPCR Master Mix (High Rox, B639273, BBI) on an ABI PRISM 3700 instrument (Foster City, CA, United States). GAPDH was used as an internal control. The primers used are as follows:

MYC-F: 5′ACT​TC-TAC​CAG​CAG​CAG​CAG 3′,

MYC-R: 5′GAG​CAG​AGA​ATC​CGA​GGA​CG 3′;

GAPDH-F: 5′TGG​GTG​TGA​AC-CAT​GAG​AAG​T 3′,

GAPDH-R: 5′TGA​GTC​CTT​CCA​CGA​TAC​CAA 3′;

MAP1LC3B-F: 5′CCG​CAC​CTT​CGA​ACA​AAG​AG 3′,

MAP1LC3B-R: 5′TCT​CCT​GGG​AGG​CAT​AGA​CC 3′.

### 2.8 Infiltration of immune cells and construction of immune characteristic subtypes

The single-sample gene set enrichment analysis (ssGSEA) algorithm is a rank-based method that defines a score representing the degree of absolute enrichment of a particular gene set in each sample (Zuo et al., 2020), With the aim of exploring the infiltration level of different immune cell populations, We obtained the immune reaction gene sets from the publication by Bindea et al. (2013), and assessed the level of immune infiltration in each sample based on the expression levels of immune cell-specific marker genes. We performed immune infiltration analysis of the ssGSEA results using the limma package (Ritchie et al., 2015) for the RA and normal groups and identified immune cells differentially expressed between these two groups. The samples were clustered according to the differentially expressed immune cells in RA using the “ConsensusClusterPlus” package in R. The parameter settings were repeated 50 times (reps = 50), and the resampling rate was 80% (pItem = 0.8). We also calculated the Pearson correlation coefficient between the expression levels of the hub gene and immune cell indices and assessed the relationship between the hub genes and immune infiltration levels.

### 2.9 Hub gene enrichment analysis and drug sensitivity analysis

GSEA was used to assess trends in the distribution of predefined gene sets to identify phenotypes associated with and interesting for hub genes (Subramanian et al., 2005). We performed GSEA on hub genes using GSEA 4.3.2 software and obtained the “c2. kegg.v7.4. symbols. gmt” and “c5. go. v7.4. symbols. gmt” from the database (Liberzon et al., 2015).

Drug sensitivity analysis was performed using the CellMiner database (https://discover.nci.nih.gov/cellminer/). The CellMiner database is primarily based on 60 cancer cell lines listed by the National Cancer Institute’s Center for Cancer Research (NCI) and is extensively described on numerous genomic and drug response platforms (Reinhold et al., 2012; Reinhold et al., 2019).

## 3 Results

### 3.1 Experimental design

**FIGURE 1:**
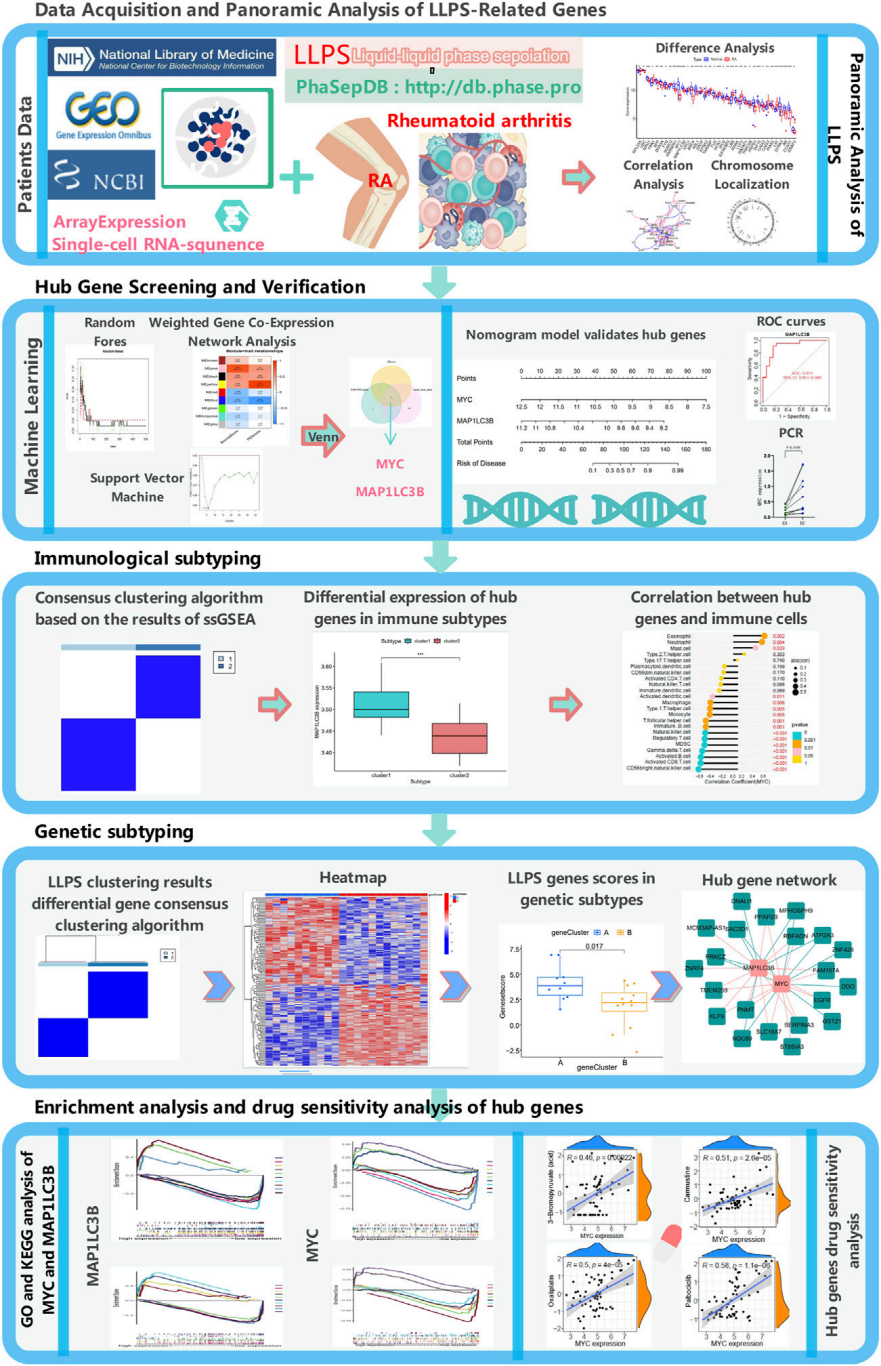
Experimental design flow chart.

As shown in Figure 1, this study screened and compared the expression of LLPS-related genes in RA and normal samples using the sample expression matrix from the GEO database. Machine learning algorithms, such as SVM, RF, and WGCNA, were applied to identify diagnostic markers, and single-cell sequencing data were used to determine the cellular localization and gene expression of the hub gene, which was subjected to PCR validation in control tissues and RA samples. We performed a series of bioinformatics analyses to validate key model genes, including functional enrichment, clinical feature-related, and immune-related analyses, drug sensitivity analysis of hub genes, and constructed molecular typing based on key genes.

### 3.2 Characterization of genes associated with LLPS in RA

**FIGURE 2:**
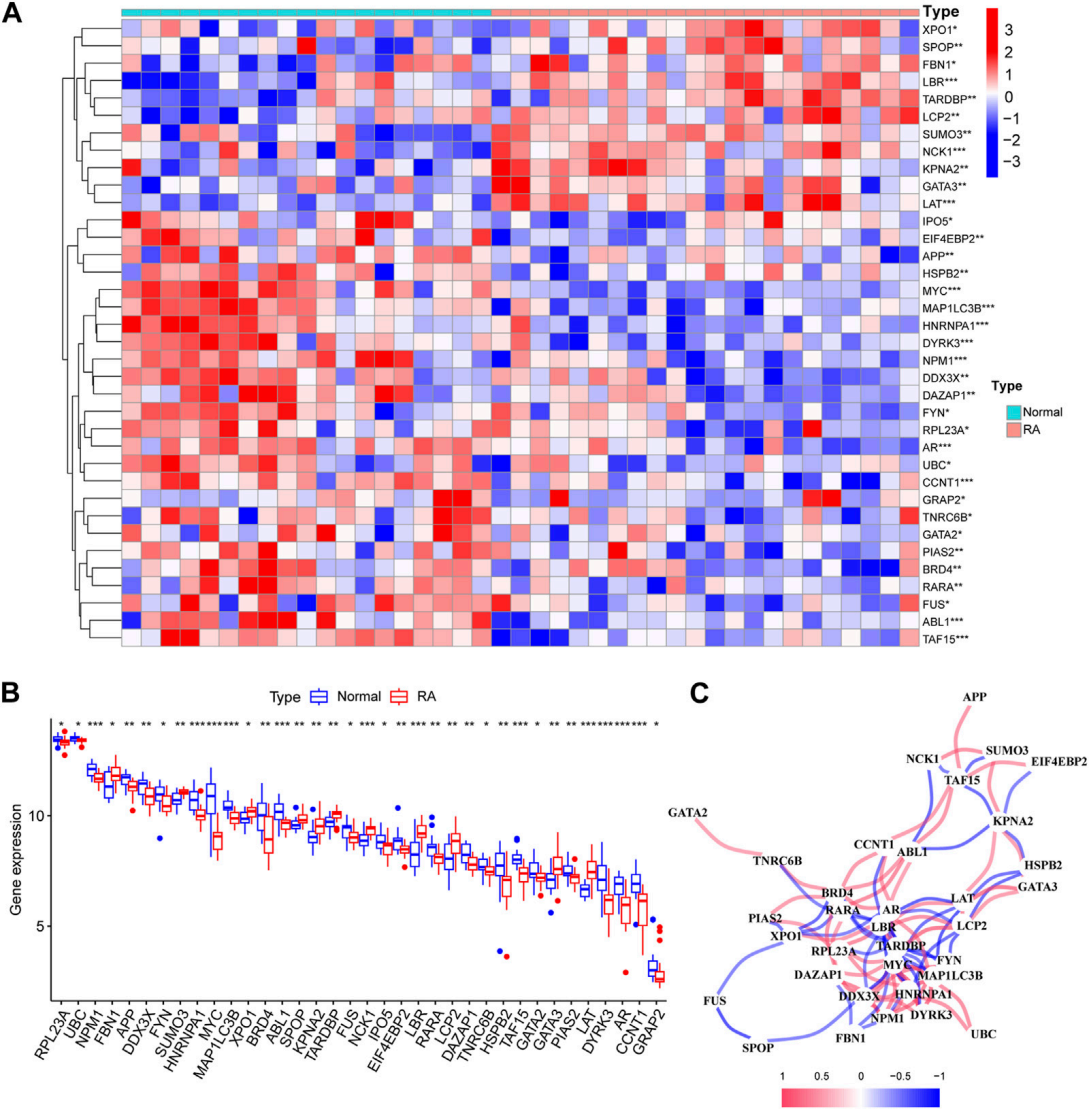
Differential analysis of liquid–liquid phase separation (LLPS)-related genes, interaction networks, and their distribution on chromosomes. **(A)** Box plot of significantly differential ex-pression of LLPS-related genes; **(B)** Heat map of differential expression of LLPS-related genes in RA; **(C)** Correlation analysis of differentially expressed LLPS-related genes, with positive corre-lations shown in red and negative correlations in blue.

To analyze the changes of phase-separated genes in the RA synovial group and to observe the effects of LLPS-related genes in patients with RA, we performed differential expression analysis of LLPS-related genes in the combined dataset. Differential analysis found 36 of 110 LLPS-related genes (RPL23A, UBC, NPM1, FBN1, APP,DDX3X, FYN, SUMO3, HNRNPA1, MYC, MAP1LC3B, XPO1, BRD4, ABL1,SPOP, KPNA2, TARDBP, FUS, NCK1, IPO5, EIF4EBP2, LBR, RARA, LCP2, DAZAP1, TNRC6B, HSPB2, TAF15, GATA2, GATA3, PIAS2, LAT, DYRK3, AR, CCNT1 and GRAP2) to be significantly differently expressed in RA patients (Figures 2A, B; Table 1), and the network between these LLPS-related genes is shown in Figure 2C. From the net-work structure map constructed based on the differential gene expression values, it can be inferred that, among the LLPS-related genes in RA, TARDBP, MYC, MAP1LC3B, HNRNPA1, NPM1, and LBR appeared to interact more closely with other genes. We show the chromosomal localization of LLPS-related DEGs in RA pathogenesis in a circle diagram Supplementary Figure S1A chromosome localization analysis showed that the LLPS-related DEGs genes were distributed on almost all chromosomes, except for chromosomes Y, 4, 7, 14, and 20.

**TABLE 1:** Differently expressed genes related to liquid-liquid phase separation.

Genes	logFC	*p*-value
MYC	−1.75	1.36E−06
TAF15	−0.80	1.36E−06
LAT	0.87	1.36E−06
MAP1LC3B	−0.56	7.33E−06
CCNT1	−1.19	1.66E−05
DYRK3	−1.16	2.23E−05
HNRNPA1	−0.71	6.02E−05
LBR	0.91	1.32E−04
AR	−1.03	1.32E−04
NCK1	0.45	2.46E−04
ABL1	−0.55	5.86E−04
NPM1	−0.40	6.44E−04
TARDBP	0.33	1.95E−03
DDX3X	−0.48	2.03E−03
KPNA2	0.50	2.60E−03
LCP2	0.74	2.85E−03
HSPB2	−0.75	3.13E−03
DAZAP1	−0.44	3.44E−03
GATA3	0.62	3.71E−03
SUMO3	0.31	4.03E−03
RARA	−0.44	4.93E−03
APP	−0.35	5.36E−03
EIF4EBP2	−0.41	6.40E−03
SPOP	0.25	6.97E−03
PIAS2	−0.30	6.97E−03
BRD4	−0.90	7.59E−03
FYN	−0.32	0.013
FBN1	0.46	0.017
XPO1	0.26	0.017
FUS	−0.30	0.017
GRAP2	−0.31	0.020
TNRC6B	−0.25	0.026
IPO5	−0.33	0.034
UBC	−0.09	0.037
GATA2	−0.28	0.039
RPL23A	−0.14	0.043

### 3.3 Molecular subtype classification of LLPS-related genes and functional enrichment analysis of key genes

**FIGURE 3:**
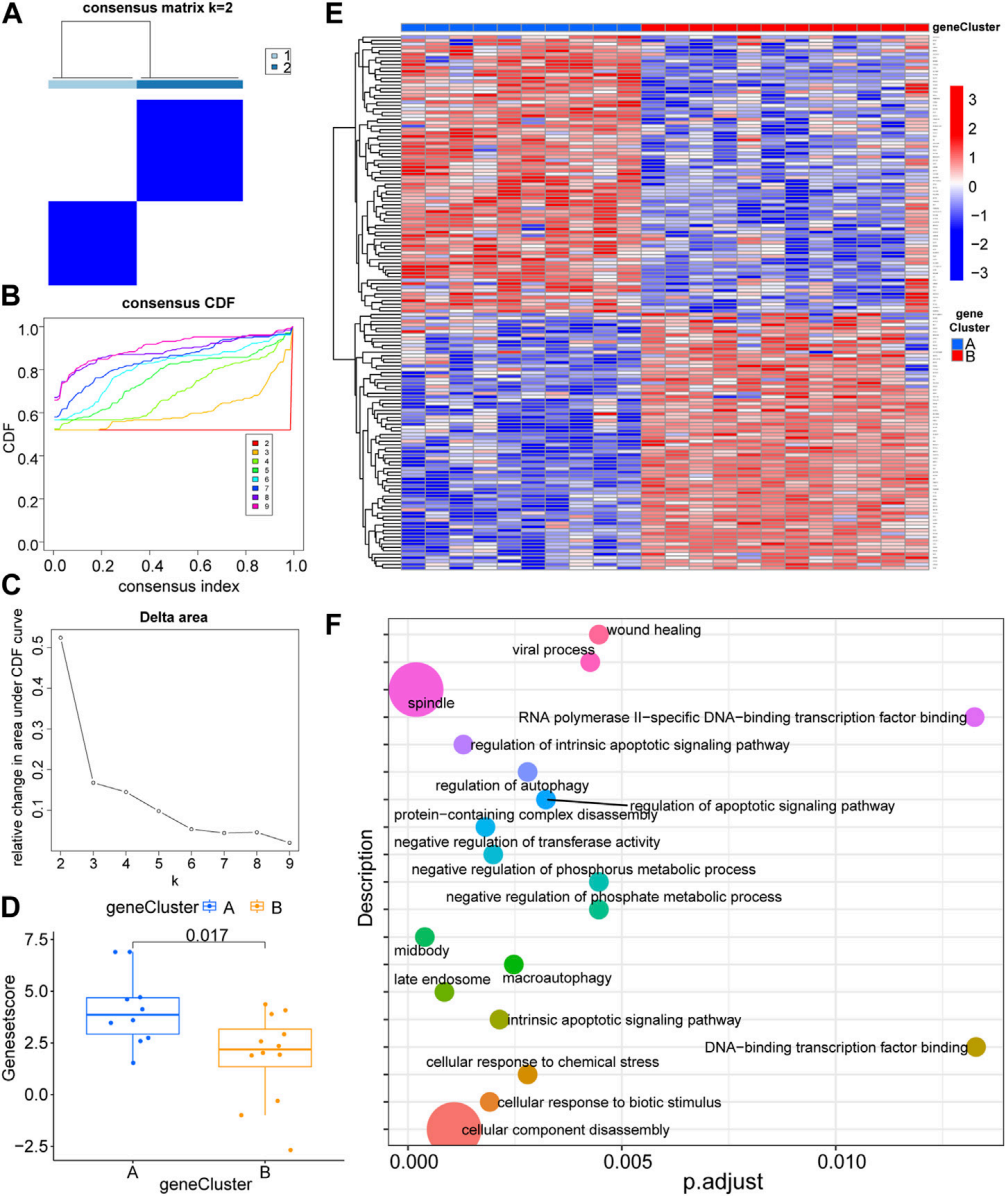
Molecular subtype consensus clustering and functional enrichment analysis of liquid–liquid phase separation-related genes. **(A)** The heat map represents the consensus matrix with a cluster count of 2. **(B)** Consensus clustering cumulative distribution function (CDF) for k = 2–9. **(C)** Relative change in area under the CDF curve for k = 2–9. **(D)** Box plot display of gene expression differences between the two groups of molecular typing. **(E)** Heat map display of differential gene expression for two groups of molecular typing. **(F)** Functional enrichment analysis of differential gene expression in two groups of molecular typing.

To further confirm the possible role of LLPS-related genes in the pathogenesis of RA, we typed the RA samples with LLPS-related differential genes (Figures 3A–C; Supplementary Figures S1B–C), and after consensus clustering, the relative increase in delta area score and stabilization after k = 3 (Figure 3C), but the matrix heat map was not sufficiently separated at k = 3 compared to at k = 2 (Figure 3A, Supplementary Figure S1B), and the slope of the Cumulative Distribution Function (CDF) curve continued to increase (Figure 3B); therefore, we chose a heat map with k = 2. The heat map was sufficiently separated under this condition, and based on LLPS differential expression, we divided the patients with RA into clusters 1 and 2 (Figure 3A, Supplementary Figure S1C) (cluster 1: n = 10, cluster 2: n = 12). The RA sample is grouped in detail in Additional file 1. Figures 3D, E show the expression of the differential genes (|log2FC| > 1, *p* < 0.05) of the two classification types in the form of box plots and heat maps, respectively. Finally, we performed functional enrichment analysis of DEGs for sub molecular typing (Figure 3F), suggesting that the LLPS genome may function through the following signaling pathways: cellular component disassembly, spindle, regulation of intrinsic apoptotic signaling pathway, protein-containing com-plex disassembly, cellular response to biotic, stimulus, negative regulation of transferase activity, intrinsic apoptotic signaling pathway, macro autophagy, regulation of autophagy, cellular response to chemical stress, regulation of apoptotic signaling pathway, viral process, negative regulation of phosphate metabolic process, negative regulation of phosphorus metabolic process, wound healing, midbody, late endosome, RNA polymerase II-specific, DNA-binding transcription factor binding, DNA-binding transcription factor binding and a box plot was used to show the overall picture of such differences.

### 3.4 Screening for hub genes by machine learning algorithm and WGCNA

**FIGURE 4:**
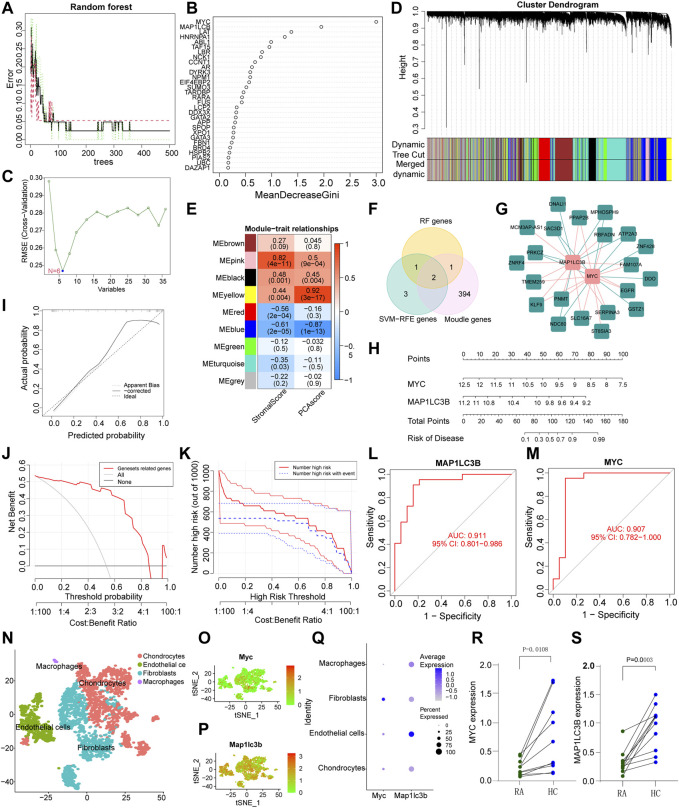
Screening of hub genes. **(A,B)**: Random forest (RF) model; **(A)** Out-of-bag error rate curve (RF model). Black line-overall accuracy; red line-sensitivity; Green line-specificity. The *X* axis indicates the number of decision trees, and the Y axis rep-resents the error rate. **(B)** Significant features identified by RF. Candidate genes are identified according to the algorithmic requirements of the RF. We selected Gini coefficients >1. **(C)** A plot of biomarkers selection via support vector machine-recursive feature elimination (SVM-RFE) algorithm. **(D)** Hierarchical clustering dendrogram of DEGs, with assigned module colors and based on the topological overlap. **(E)** Heatmap of the correlation of modular signature genes with status and liquid–liquid phase separation (LLPS) database types in RA patients. Each cell contains the Pearson correlation coefficient and *p*-value, with blue indicating a negative correlation and red a positive correlation. **(F)** Multi-algorithm results overlay to screen RA pathogenicity models from LLPS-related genes. **(G)** Differential gene and hub gene association network analysis from LLPS differential genotyping. **(H)** Establishment of a nomogram model based on multiple algorithm screening. **(I)** Predictive robustness of the nomogram model based on the calibration curve. **(J)** Decisions based on the nomogram model may benefit RA patients. **(K)** The clinical impact of the nomogram model, as assessed by the clinical impact curve. **(L,M)** ROC curves of MAP1LC3B and MYC in the nomogram model. **(N)** A scatter plot showing the different cellular fractions of the arthritic knee synovium by color. **(O)** and **(P)** show the expression of MYC and MAP1LC3 in all cells, respectively. **(Q)** The average expression and percentage expression of MYC and MAP1LC3 in the four types of RA synovial cells. **(R,S)** MYC and MAP1LC3 expression levels in RA and adjacent normal tissue (*p < 0.05).

In order to predict the diagnostic biomarkers associated with LLPS that have the most important impact on the development of RA, we applied two different machine algorithms and WGCNA. We first built an RA prediction model for LLPS-related genes using the RF algorithm and identified four feature subsets: MYC, MAP1LC3B, LAT, and HNRNPA1, the results are shown in Figures 4A, B. As shown in Figure 4A, the error rate decreased as the number of trees increased. When the number of trees reached 100, the error rate started to stabilize at 0.05; that is, 95% of the samples were corrected by RF classification. Figure 4B shows that the main risk genes in the model with a Gini coefficient greater than 1 were MYC, MAP1LC3B, LAT, and HNRNPA1. The SVM-RFE algorithm was used to identify feature variables associated with LLPS in RA, and the results showed that the classifier produced the least error when the number of features was six. The feature variables identified were MYC, LAT, MAP1LC3B, TAF15, CCCT1, and ABL1 (Figure 4C). WGCNA (Figures 4D, E) was conducted using transcriptome profiling data, PCA scores, and stromal scores of the samples. The blue module was identified as having a higher correlation with the PCA score (R > 0.3, *p* < 0.0001). Using <0.01 as the threshold of the *p*-value for univariate Cox regression, 97 genes with gene significance >0.5 and module membership >0.7 from the blue module were identified as promising candidates related to the prognosis of patients with RA. Finally, the common genes obtained by overlapping the screening genes of SVM-RFE, RF, and WGCNA were further analysed (Figure 4F). To further confirm the correlation between hub genes and LLPS, we performed correlation analysis of submolecular typing of differential and hub genes based on the expression values of differential and hub genes using Limma package and visualized the network relationships using Cytoscape software (Figure 4G). The correlation of sub molecular typing of differential genes and hub genes is shown in Additional file 2. To better evaluate the RA risk assessment model constructed based on the LLPS-related genes screened by multiple algorithms, we constructed a nomogram model based on the expression of MYC and MAP1LC3B of the model genes (Figure 4H), and the calibration plot (Figure 4I) revealed that the nomogram was well calibrated. The model developed by LLPS-related genes is always at the top of the DCA curve (Figure 4J), indicating that our nomogram model has a high predictive performance. The clinical impact curve (Figure 4K) showed that the number of high-risk patients screened by the model far exceeded the number of high-risk patients experiencing the event, indicating that patients with RA clearly benefit from decisions based on this nomogram. The AUC of the LLPS-related genes, MAP1LC3B and MYC, in the model were 0.911 and 0.907, respectively (Figures 4L, M), indicating that the nomogram was highly predictive. To further validate the accuracy and sensitivity of the hub genes screened in this study in RA, we verified the expression of the hub genes in the GSE55457 dataset, and further ROC analysis of the MAP1LC3B and MYC genes in the GSE55457 dataset showed that the AUC of the two genes were 0.785 and 0.815, respectively (Supplementary Figures S1D, E). These analyses suggest that the MAP1LC3B and MYC genes have high diagnostic and therapeutic accuracy for RA. Differential analysis of the MYC and MAP1LC3B genes in the GSE55457 dataset showed that the expression of MYC and MAP1LC3B was significantly lower in RA patients than in normal control patients (Supplementary Figures S1F,G), which is consistent with the results of previous studies.

### 3.5 High cellular heterogeneity in RA tissues discovered by scRNA-sequencing

To understand the cellular heterogeneity of synovial tissues and the cellular heterogeneity of hub genes, we first selected scRNA-seq data of mice with serum transfer inflammatory arthritis from the GEO database, and using the “singleR” R package, we classified the cells into four major categories labeled as chondrocytes, endothelial cells, fibroblasts, and macrophages (Figure 4N). Subsequently, we analyzed the expression of MYC and MAP1LC3B in cell clusters in RA synovial tissue, and in agreement with our RNA transcriptome data results, MYC showed low expression in the 4-cluster cell population, while inconsistently, MAP1LC3B showed high expression in the 4-cluster cell population, which requires further studies and support from the literatur (Figures 4O–Q). MYC expression was higher in fibroblasts compared to the rest of the cells (Figures 4O, Q). To further demonstrate the transcriptome regulation of fibroblasts in synovial tissues of RA patients, we performed UMAP analysis on scRNA-seq datasets of human RA tissues and classified fibroblasts into five classes. To further name these type 5 cells, we filtered the top five marker genes in each cell type according to log|FC| and drew a heatmap of gene expression (Supplementary Figure S2A), and named these type 5 cells as SPRF1^+^ fibroblasts, COMP^+^ fibroblasts, RYR3^+^ fibroblasts, PRG4^+^ fibroblasts, and SPARC^−^ fibroblasts, respectively (Supplementary Figure S2B). Analysis of MYC and MAP1LC3B expression in different types of fibroblasts showed that MYC expression in fibroblasts was lower than that of MAP1LC3B, consistent with previous single-cell results in mice metastatic arthritis (Supplementary Figures S2C–E). qRT-PCR showed that the expression of MYC and MAP1LC3B in RA synovial tissues was significantly lower than that in the normal control synovial tissues (Figures 4R, S).

### 3.6 Correlation analysis of hub genes and immune cell infiltration

**FIGURE 5:**
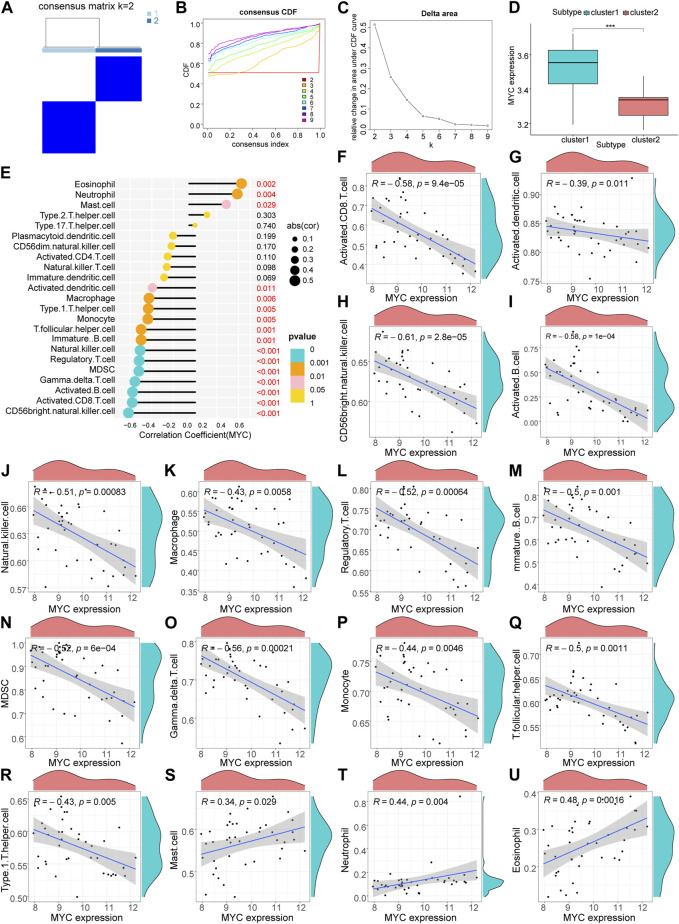
Hub gene MYC immune infiltration characteristics. **(A)** Matrix heat map, rows and columns are samples. **(B)** Cumulative distribution function curve showing different k values. **(C)** Delta area score of the Cumulative Distribution Function curve. **(D)** Differential ex-pression of hub gene: MYC in immune subtypes (***p < 0.001). **(E–U)** Correlation analysis of MYC and immune cells.

**FIGURE 6:**
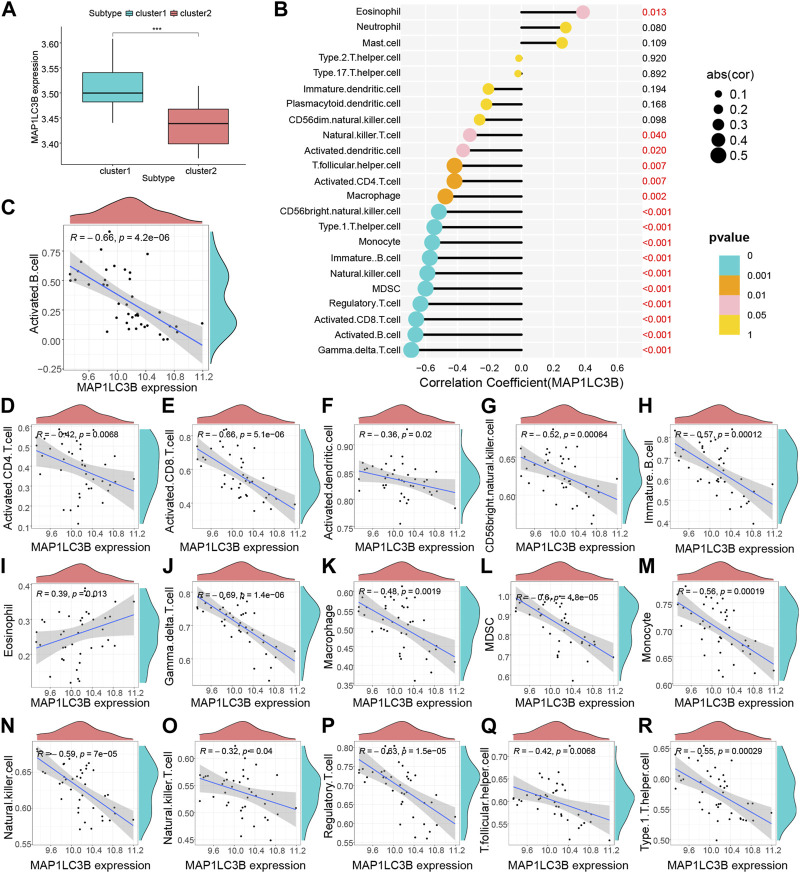
Hub gene MYC immune infiltration characteristics. **(A)** Differential analysis of immune cells between the RA group and control group (***p < 0.001, **p < 0.01, *p < 0.05, ns: no significant difference). **(B–R)** Correlation analysis of MYC and immune cells.

Based on the results of the ssGSEA analysis (Additional file 3), we used consensus clustering to classify RA patients into different immune subtypes (Figures 5A–D; Supplementary Figures S1H–J). Figure 5C shows that the delta area score of the CDF curve tends to increase at k = two to nine and is relatively stable after k = 5, but the matrix heat map is not completely separated at k = 3, 4 and 5 (Supplementary Figures S1H–J). At k = 2, the slope of the CDF curve (Figure 5B) was minimal, and the matrix heat map was fully separated (Figure 5A). Therefore, we divided patients with RA into two subtypes with distinct immune signatures (cluster 1, n = 22; cluster 2, n = 19). The correlation between the hub gene, *MYC, MAP1LC3B* and immune signature isoforms revealed that *MYC, MAP1LC3B* were significantly overexpressed in the “cluster1” group (Figure 5D; Figure 6A), suggesting that hub genes may be immunologically relevant in the pathogenesis of RA. To study the potential correlation between the hub genes *MYC* and *MAP1LC3B* and the efficacy of immunotherapy, we analyzed the correlation between the hub genes and the immune microenvironment and found that the hub genes *MYC* and *MAP1LC3B* had a significant positive and negative correlation with the degree of infiltration of various innate and acquired immune cells(|cor|>0.4) (Figures 5E–U, Figures 6B–R). The results of the correlation between hub genes and immune cells are presented in Additional file 4.

### 3.7 Validation of immune function of hub genes in LLPS genotyping of RA

**FIGURE 7:**
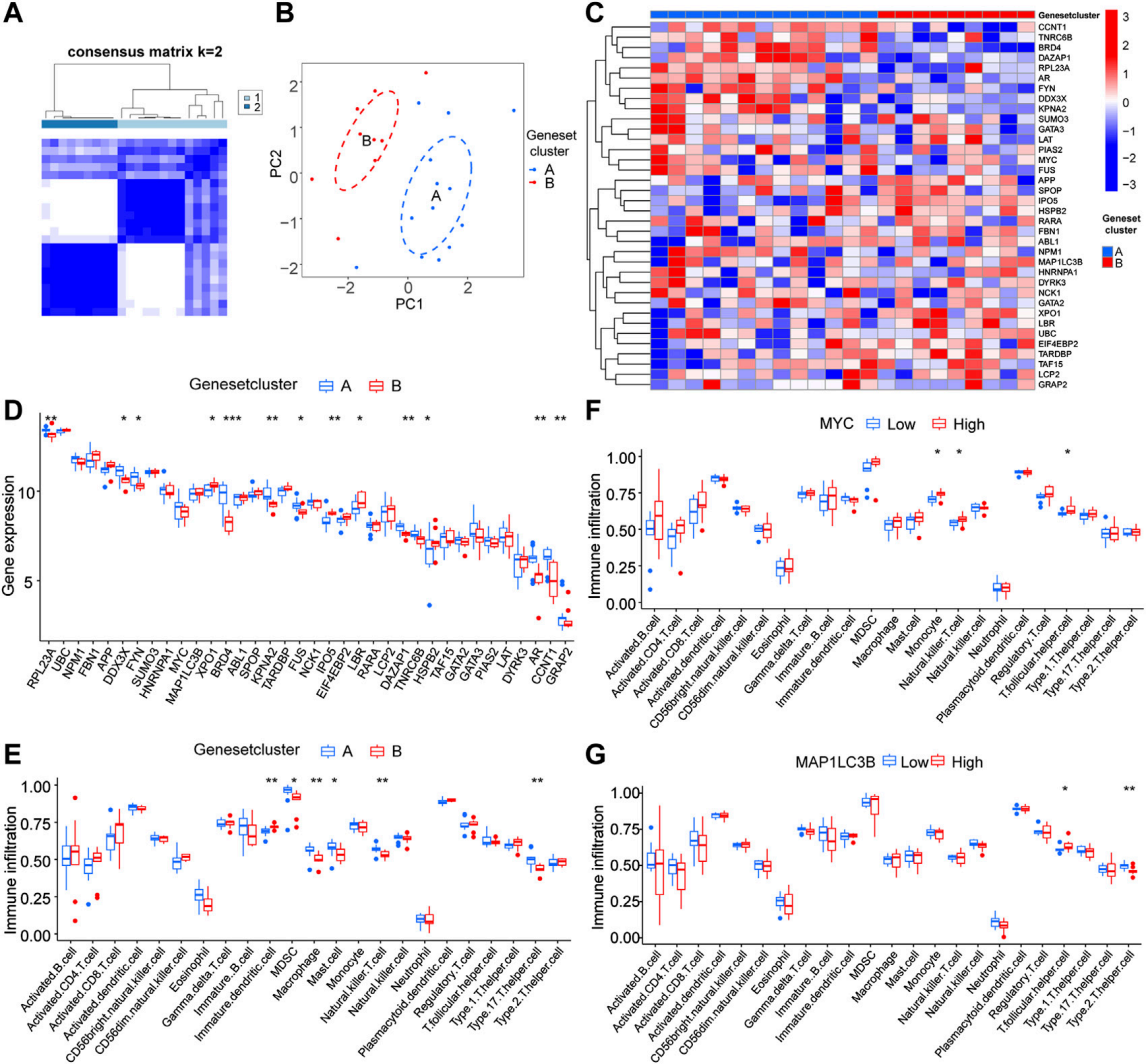
Validation of immune function of hub genes in genotyping **(A)** Matrix heat map, rows and columns are samples. **(B)** Principal component analysis (PCA) of different immune signature subtypes; blue indicates cluster 1, red indicates cluster 2. **(C)** Heat map of differential gene expression associated with liquid–liquid phase separation in RA sub molecular typing. **(D)** Significant DEGs in clusters A and B. **(E)** Significantly different immune infiltrated cells in the two groups A and B. **(F,G)** Correlation analysis between the expression levels of hub genes MYC and MAP1LC3B and immune cells.

To further confirm the hub gene functions, we performed a clustering analysis within the RA sample group based on LLPS-related DEGs (Figure 7A). All samples were divided into two RA molecular subtypes (cluster A, n = 13; cluster B, n = 9), and the PCA results showed a high-quality separation of the two RA molecular subtypes (Figure 7B). We also showed LLPS-related differential gene expression in the RA sub-molecular typing base using a heat map (Figure 7C). Figure 7D shows the differences in gene ex-pression in the two molecular subtype classifications, while Figure 7E shows differences in immune infiltration in immature dendritic cells, myeloid-derived suppressor cells, macrophage, mast cells, natural killer cells, and type 17 helper cells in the two subtypes.

To study the potential correlation between the hub genes, MYC and MAP1LC3B, and the efficacy of immunotherapy, we analyzed the correlation between hub genes and the immune microenvironment and found that MYC was significantly correlated with the degree of infiltration of various innate and acquired immune cells, such as monocytes, natural killer T cells, and T.follicular.helper.cell (Figure 7F) and MAP1LC3B had a significant correlation with the degree of infiltration of various innate and acquired immune cells, such as T follicular helper cell and Type 2 T helper cell (Figure 7G).

### 3.8 Hub genes functional analysis and drug sensitivity analysis

**FIGURE 8:**
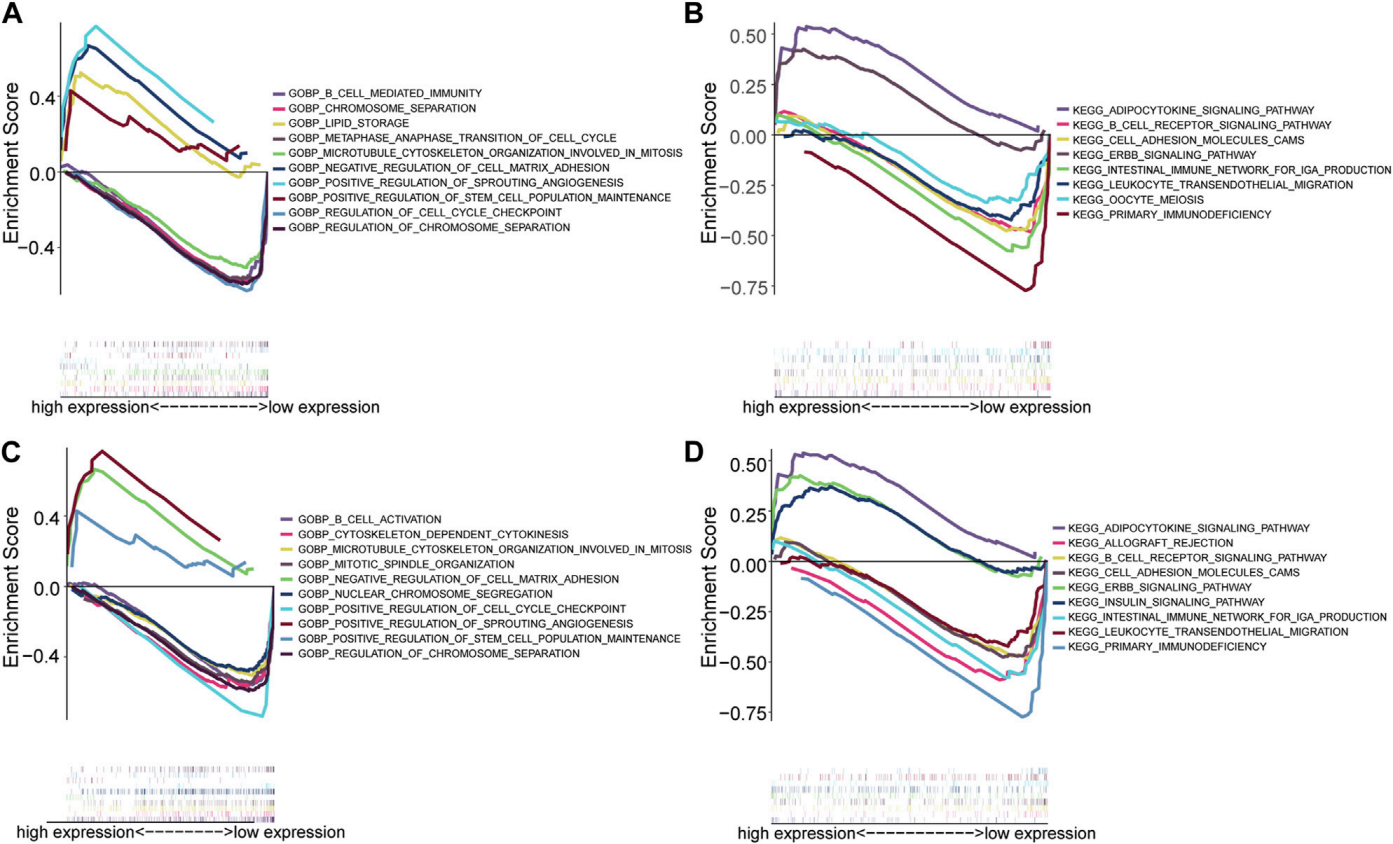
Hub gene single gene enrichment analysis. **(A)** The GO enrichment analysis of MYC. **(B)** The KEGG enrichment analysis of MYC. **(C)** GO enrichment analysis of MAP1LC3. **(D)** KEGG enrichment analysis of MAP1LC3.

Single gene GSEA enrichment analysis revealed that the GO enrichment function of MYC (Figure 8A) included positive regulation of sprouting angiogenesis, regulation of chromosome separation, b cell mediated immunity, positive regulation of stem cell population maintenance, negative regulation of cell matrix adhesion, lipid storage, chromosome separation, metaphase anaphase transition of cell cycle, microtubule cytoskeleton organization involved in mitosis, regulation of cell cycle checkpoint. The KEGG enrichment functions of MYC (Figure 8B) include primary immunodeficiency, ErbB signaling pathway, adipocytokine signaling pathway, cell adhesion molecules cams, B cell receptor signaling pathway, leukocyte trans endothelial migration, intestinal immune network for iga production, oocyte meiosis. The GO enrichment functions of MAP1LC3B (Figure 8C) include microtubule cytoskeleton organization involved in mitosis, mitotic spindle organization, positive regulation of cell cycle checkpoint, regulation of chromosome segregation, cytoskeleton-dependent cytokinesis, nuclear chromosome segregation, B cell activation, positive regulation of stem cell population maintenance, negative regulation of cell matrix adhesion, and positive regulation of sprouting angiogenesis. The KEGG enrichment functions of MAP1LC3B (Figure 8D) include primary immunodeficiency, ErbB signaling pathway, adipocytokine signaling pathway, cell adhesion molecules cams, intestinal immune network for IgA production, leukocyte trans endothelial migration, B cell receptor signaling pathway, allograft rejection, and insulin signaling pathway. The detailed enrichment results of the two hub genes are shown in Additional file 5.

**FIGURE 9:**
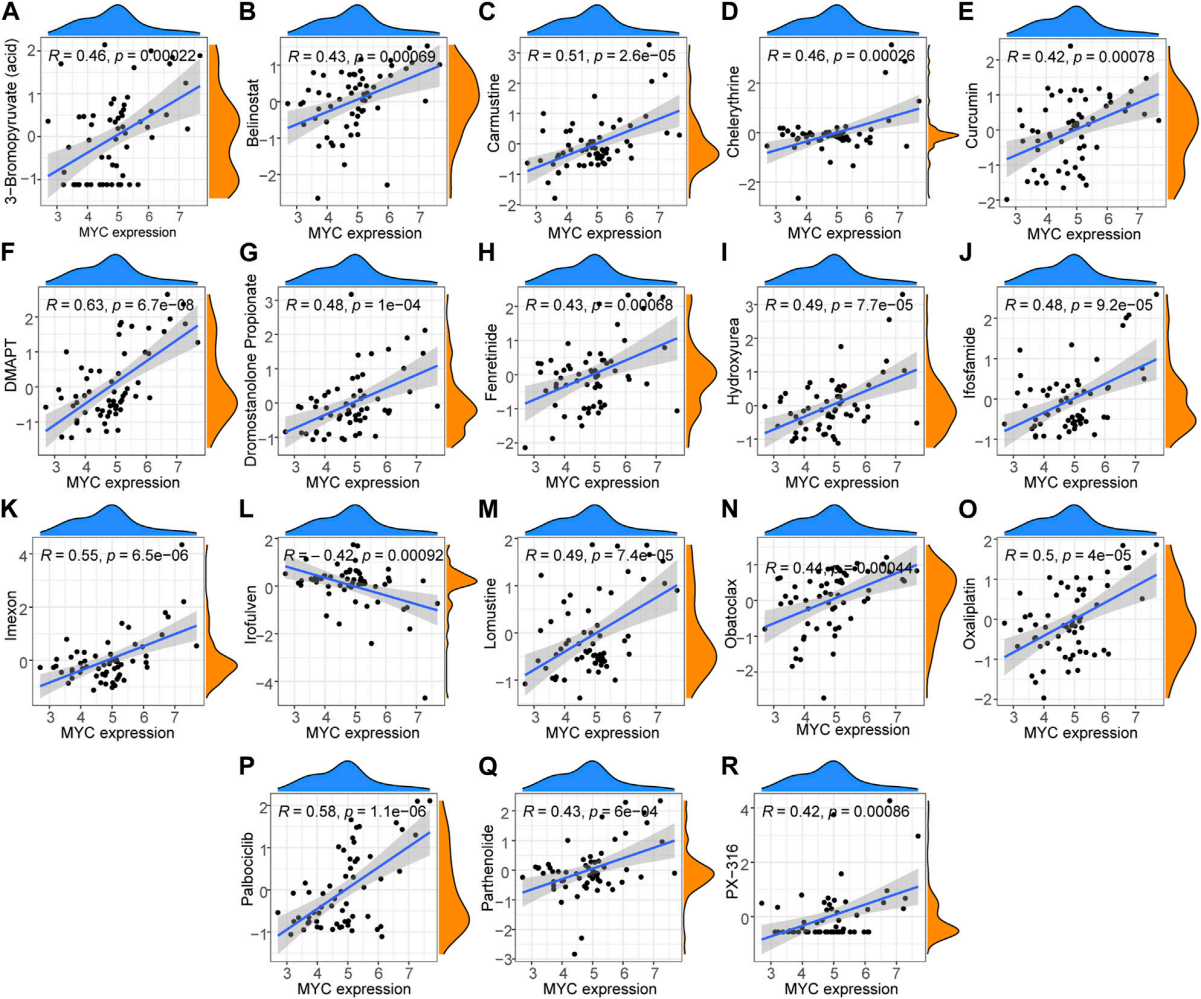
Hub gene drug sensitivity analysis. **(A–K, M–R)** Expression of MYC was correlated with the sensitivity for DMAPT, palbociclib, imexon, carmustine, oxaliplatin, lomustine, hydroxyurea, ifosfamide, dromostanolone propionate, 3-bromopyruvate (acid), chelerythrine, fenretinide, obatoclax, parthenolide, fenretinide, belinostat, curcumin, PX-316, and **(L)** irofulven were positively correlated with drug sensitivity but was negatively correlated with that for irofulven.

Drug sensitivity analysis (Figures 9A–R) showed that DMAPT, palbociclib, imexon, carmustine, oxaliplatin, lomustine, hydroxyurea, isocyanide, dromostanolone propionate, 3- bromopyruvate (acid), chelerythrine, fenretinide, obatoclax, parthenolide, fenretinide, belinostat, curcumin and PX-316 were each positively correlated with MYC expression, while irofulven was negatively correlated with MYC expression.

## 4 Discussion

RA is the most common inflammatory arthritis and a major cause of disability. Immune mechanisms are at the forefront of RA pathogenesis (Firestein, 2003). According to in-depth studies of its genomics and based on its pathogenesis, specific prophylactic measures can be designed to suppress synovitis and arthritis. LLPS is becoming a widely accepted theory explaining the spatial and temporal control of complex intracellular biochemical reactions by membraneless organelles (Banani et al., 2017; Shin and Brangwynne, 2017). These condensates, the so-called MLOs, represent distinct liquid phases that are selectively enriched in certain macromolecules and fulfill essential cellular functions under normal conditions and in response to stress (Alberti, 2017; Li et al., 2018; Alberti et al., 2019; Pancsa et al., 2019). Emerging studies (Su et al., 2016; Sheu-Gruttadauria and MacRae, 2018; Zhang et al., 2018; Nozawa et al., 2020) have shown that LLPS is involved in diverse pathological and physiological processes, such as adaptive and innate immune signaling, stress granule assembly, heterochromatin formation, transcription, miRISC assembly, and autophagy, LLPS also plays a role in the development of cancer and neurodegenerative and inflammatory diseases. However, the importance of LLPS in RA has not yet been fully elucidated. This study aimed to investigate the effects of LLPS-related genes on RA.

In this study, we performed a multi-machine algorithmic analysis of RA multiomics data to determine the expression, heterogeneity, and immunological value of LLPS-related genes in RA. We first analyzed the phenotypic characteristics of LLPS-related genes in RA, focusing on the distribution of LLPS-related GESs in RA and on chromosomes, and the structure of the interaction network constructed based on gene expression values. By applying two algorithms widely used in the fields of pattern recognition and machine learning to predict the impact of LLPS-related genes on the pathogenesis of RA, simultaneous WGCNA identified blue modules that were highly associated with LLPS in RA. By crossing the genes in this module with two previous machine algorithm model genes, these genes (including *MYC* and *MAP1LC3B*) were identified as key genes associated with the regulation of LLPS in RA. Meanwhile, we constructed nomogram models for RA risk assessment model expression (*MYC* and *MAP1LC3B*), and multiple validations of the nomograms demonstrated that the nomogram models have a strong clinical predictive power for RA. One study (Van Raemdonck et al., 2021) found that IL-34 arthritic joint *C-MYC (3x)* was upregulated to trigger increased glucose uptake compared with that in non-arthritic mice; however, blood glucose concentrations were downregulated in arthritic mice 15 and 30 min after glucose injection. In addition, MYC may also promote an increase in glycolysis-dependent oxidative phosphorylation during RA osteoclast development (Sakuraba et al., 2022). A osteoclast development. It has also been suggested that MYC may be an important bridge between inflammation and heterotopic ossification in ankylosing spondylitis (Jin et al., 2023). In a study (Fang et al., 2021) on the association between tumor grade and LLPS-related genes, the LLPS-related genes *E2F* and *MYC* may be important determinants of survival in high-risk groups. The hub gene of interest in our study, *MAP1LC3B*, is an important link in biological autophagy. Mitotic receptors with a conserved MAP1LC3/LC3 interacting region mark damaged mitochondria for recruitment to the autophagy machinery through direct interaction with LC3 and other ATG proteins (Chen et al., 2016). In a study of ischemic stroke, MAP1LC3B was identified as a diagnostic marker for ischemic stroke and was highly expressed in the disease group, and MAP1LC3B was also found to be a potential biomarker for docosahexaenoic acid sensitivity in colorectal cancer cells (Chen et al., 2021).

Based on the results of the ssGESEA analysis, we used consensus clustering to classify RA patients into different immune subtypes, and the model hub genes *MYC* and *MAP1LC3B* were significantly highly expressed in the immune subtype “cluster 1” group. We also performed correlation analysis of the hub genes with the degree of infiltration of various immune cells and sensitivity analysis with drugs to provide a basis for elucidating the pathogenic mechanism and immunotherapy of LLPS-related hub genes in RA. MYC was found to be associated with CD56bright natural killer cell, CD8 + T cell, activated B cell, gamma delta T cell, Myeloid-derived suppressor cell (MDSC), regulatory T cell, natural killer cell, immature B cell, T follicular helper cell was significantly negatively correlated with eosinophil, neutrophil immune cells and positively correlated with eosinophil, neutrophil immune cells. MAP1LC3B is significantly negatively correlated with gamma delta T cell, activated B cell, activated CD8 T cell, regulatory T cell, MDSC, natural killer cell, immature B cell, monocyte, type 1 T helper cell, CD56bright natural killer cell and other immune cells were significantly negatively correlated.

*MYC* proteins are master regulators of cellular programs (Dhanasekaran et al., 2022). Previous studies (Guo et al., 2022) have shown that *MYC* is involved in immunopathological processes mediated by B cells and T cells through various mechanisms, during the first division of activated CD8 + T cells, *cBAF* and *MYC* are often asymmetrically co-allocated to the two daughter cells, daughter cells with high MYC and cBAF expression showed a cell fate towards T cells, whereas daughter cells with low MYC and cBAF expression preferentially differentiated towards T cells. Di Pietro showed that deletion of Bmi1 restored *c-Myc* expression in B cells and increased the quality of antibodies (Di Pietro et al., 2022). Wang et al. (2017) found that MYC protein-positive diffuse large B-cell lymphomas are characterized by highly activated B-cell receptor signaling. It is well known that MDSCs contribute to tumor immune evasion. MDSCs not only significantly promoted tumorsphere formation, cell colony formation, and cancer stem cell accumulation, but also enhanced the expression of stemness biomarkers NANOG and c-MYC in epithelial ovarian cancer cells during co-culture (Li et al., 2020). It was shown that activated T cells and natural killer cells infiltrating the RA synovium can induce apoptosis in RA synovial cells through Fas/Fas-l interactions (Asahara et al., 1996). In addition, MYC was found to be significantly negatively correlated with follicular helper T cells in atrial fibrillation (Liu et al., 2021b). Myc overexpression leads to an increase in liver-infiltrating neutrophils, and this increase can inhibit tumorigenic liver growth by suppressing neutrophil differentiation through angiogenesis inhibitors or morpholino knockdown (Zhao et al., 2016). In addition, *MAP1LC3/LC3* could be involved in autophagy-mediated B and T cell immune responses through various mechanisms. In oral cancer and hepatocellular carcinoma, MAP1LC3B expression was significantly and positively correlated with the number of MDSCs and monocyte density, respectively (Chen et al., 2018; Wu et al., 2018).

Biological characterization of LLPS-related hub genes using single-gene GSEA enrichment analysis showed that the GO functions of MYC and MAP1LC3B are mainly enriched in vascular, stromal, and immune regulation, cell cycle, and immune regulation. KEGG functional enrichment analysis of these two hub genes showed that they are mainly involved in immune regulation and signaling. These results suggest that the LLPS-related hub genes play an integral role in RA.

Hub genetic drug sensitivity analysis showed that although only *MYC* had results with 19 drugs, some drugs are now used in the clinical treatment of RA. Moreover, RA in patients improved when these drugs were used in the treatment of patients with cancer RA. Improvement in RA in breast cancer patients treated with palbociclib has been reported (Murakami et al., 2021). Low-dose hydroxyurea is safe and effective in the treatment of RA (Ehrlich et al., 1995). Chelerythrine ameliorates RA by modulating the AMPK/mTOR/ULK-1 signaling pathway (Cai et al., 2022). Fenretinide exhibited anti-inflammatory/anti-arthritic properties (Paul et al., 2016). Parthenolide inhibited the proliferation of fibroblast-like synoviocytes *in vitro* (Parada-Turska et al., 2008). Furthermore, a review evaluated the therapeutic efficacy and changes in inflammatory parameters of curcumin in RA and reported various possible mechanisms of curcumin in the treatment of RA (Pourhabibi-Zarandi et al., 2021). Our bioinformatics algorithm was partially validated in terms of the drug sensitivity of key genes, providing the basis for our next study, focusing on investigating the role of LLPS-related genes in the pathogenesis and clinical treatment of RA. It also provides a guiding direction for future research and clinical treatments. However, this study has certain limitations and did not validate the role of LLPS in RA in molecular biology. Therefore, further studies are necessary to establish the correlation between RA and LLPS.

## 5 Conclusion

In conclusion, the present study demonstrated that the expression of LLPS-related genes MYC and MAP1LC3B was downregulated during the development of RA inflammation and was accompanied by the accumulation of immune cells such as CD56bright natural killer cells, gamma-delta T cells, MDSCs, regulatory T cells and immature B cells, which are potential targets for RA immunotherapy. However, we lack corresponding clinical cohort to evaluate the practicality and accuracy of this signature. We will improve it in the future.

## Data Availability

The datasets presented in this study can be found in online repositories. The names of the repository/repositories and accession number(s) can be found in the article/Supplementary Material.

## Supplementary material

SUPPLEMENTARY FIGURE S1
**(A)** Chromosomal localization of differ-entially expressed liquid–liquid phase separation (LLPS)-related genes. **(B)** The heat map of the consensus matrix with a cluster count of 3 in the molecular subtype consensus clustering of LLPS genes. **(C)**Tracking plot at k = 2–9 in the consensus clustering of molecular subtypes of the LLPS gene. **(D,E)** ROC curves of MYC and MAP1LC3B in the GSE55457 dataset. **(F,G)** MYC and MAP1LC3 expression levels in RA and adjacent normal tissue in the GSE55457 dataset (**p* < 0.05, ***p* < 0.01). **(H–J)** Matrix heatmap of the consensus clustering of the hub gene MYC immune signature subtypes, rows and columns are samples.

SUPPLEMENTARY FIGURE S2
**(A)** Heatmap of expression levels of significantly expressed genes in 5 fibroblasts types, with purple as low expression genes and yellow as high expression genes. **(B)** A scatter plot showing the different fibroblasts of the RA knee synovium by color. **(C,D)** show the expression of MYC and MAP1LC3 in all fibroblasts, respectively. **(E)** The average expression and percentage expression of MYC and MAP1LC3 in the five types of RA fibroblasts.

## Abbreviations

RA, Rheumatoid arthritis; LLPS, liquid–liquid phase separation; MLOs, membraneless organelles; DEGs, differentially expressed genes; GEO, Gene Expression Omnibus; RF, Random Forest; SVM, Support Vector Machine; WGCNA, Weighted Gene Co-Expression Network Analysis; SVM-RFE, Support vector machine recursive feature elimination; PCA, principal component; ROC, receiver operating characteristic; AUC, area under curve; DCA, decision curve analysis; ScRNA, small cytoplasmic RNA; qRT-PCR, quantitative real-time polymerase chain reaction; ssGSEA, single-sample gene set enrichment analysis; CDF, Cumulative Distribution Function; AUC, areas under the ROC curve; t-SNE, t-distributed stochastic neighbor embedding; MDSC, Myeloid-derived suppressor cell.
