# Supplementary figures and images for: Single-cell sequencing and transcriptome analyses in the construction of a liquid–liquid phase separation-associated gene model for rheumatoid arthritis
Source: Front Genet. 2023 Oct 25;14:1210722. doi: 10.3389/fgene.2023.1210722 (PMC10634374; doi:10.3389/fgene.2023.1210722)

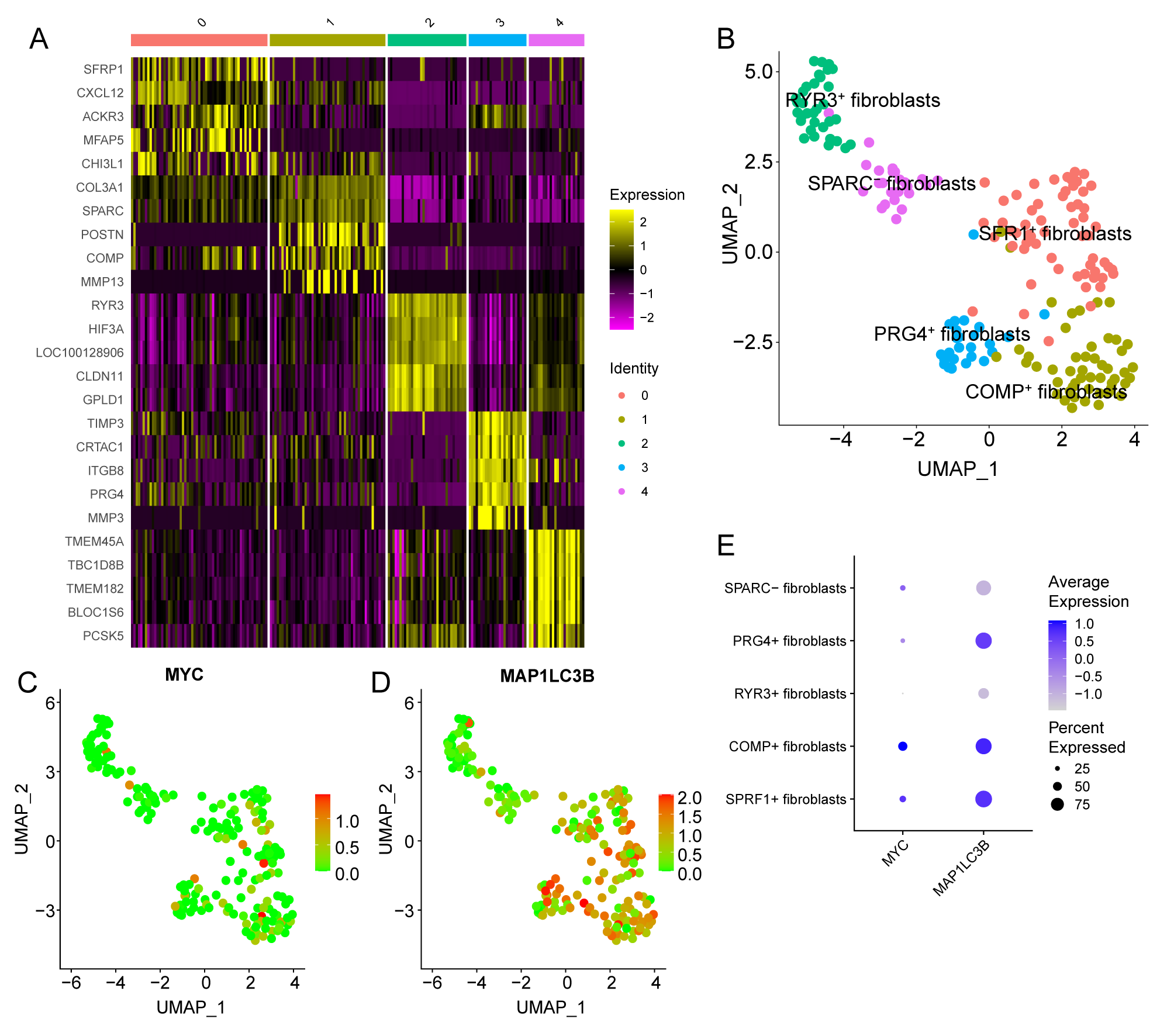

Supplement: Supplementary file 3 [file Image2.tif]

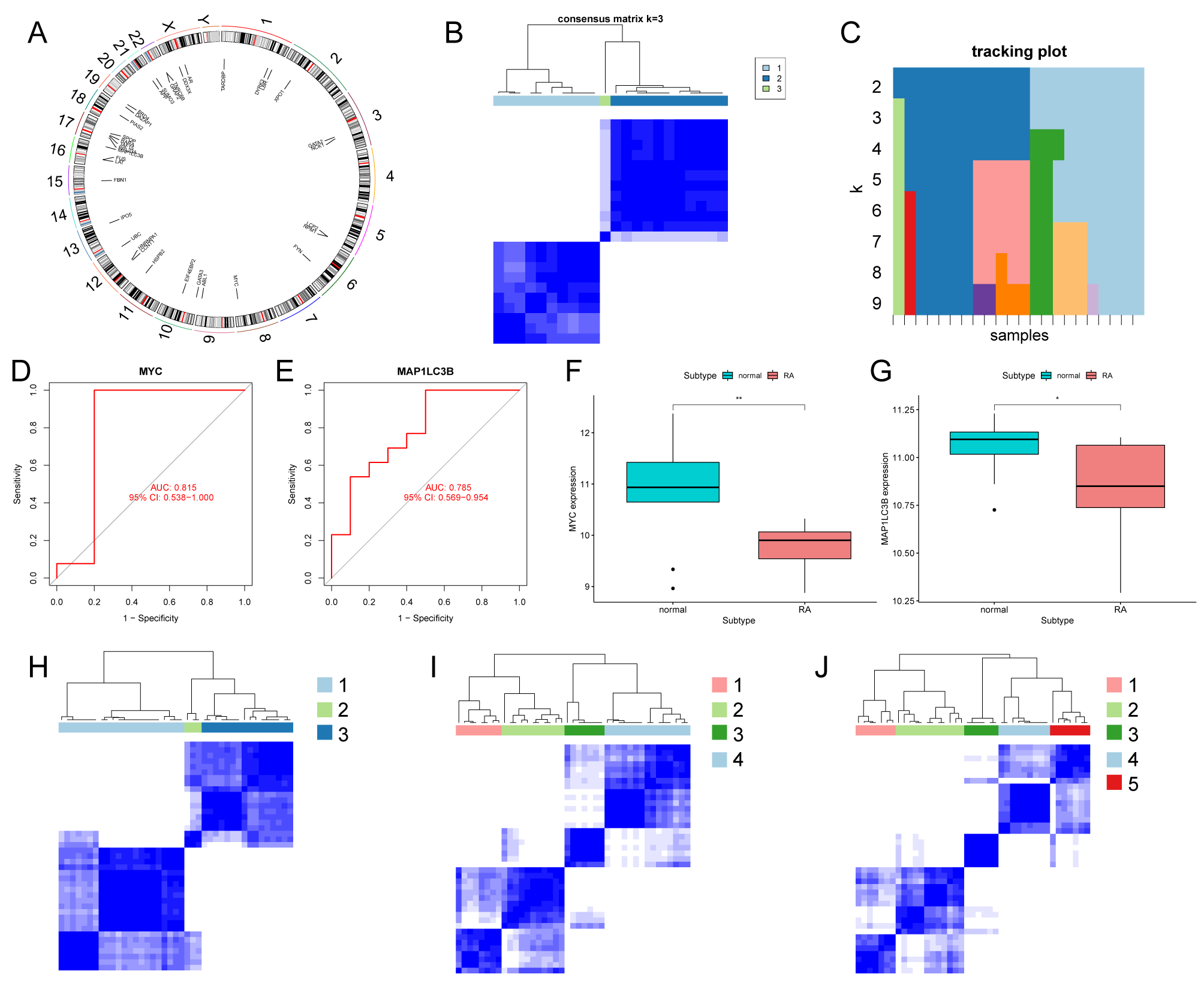

Supplement: Supplementary file 4 [file Image1.tif]
